# Editorial: Towards the Identification of Useful Genes for *Prunus* Breeding

**DOI:** 10.3389/fpls.2022.945476

**Published:** 2022-06-07

**Authors:** Elena Zuriaga, Ryutaro Tao, Carlos Romero

**Affiliations:** ^1^Centro de Citricultura y Producción Vegetal, Instituto Valenciano de Investigaciones Agrarias, Valencia, Spain; ^2^Laboratory of Pomology, Graduate School of Agriculture, Kyoto University, Kyoto, Japan; ^3^Department of Biotechnology and Plant Breeding of Cultivated Species, Instituto de Biología Molecular y Celular de Plantas, Consejo Superior de Investigaciones Científicas—Universitat Politécnica de València, Valencia, Spain

**Keywords:** *Prunus*, breeding, stone fruits, marker assisted selection, QTLs

Stone fruits (*Prunus* genus, Rosaceae family) are cultivated worldwide and constitute an economically relevant group within woody crop species. Conventional breeding in *Prunus*, mainly based on phenotyping, is rapidly giving way to a more efficient selection assisted by molecular markers. QTL mapping and the identification of genes underlying agronomic traits control are the driving force for this transition. Literature available in this field was thoroughly reviewed by Aranzana et al. (2019) but recently new contributions have seen the light of day. For instance, studies regarding fruit skin color regulation in Japanese plum (Fiol et al., 2021), low-temperature tolerance in Japanese apricot (Ding et al., 2021) and powdery mildew resistance in peach (Marimon et al., 2020). Current breeding efforts in stone fruits are primarily focused on fruit yield and quality, pest and disease resistances, and environmental adaptation. However, our knowledge on the genetic control of many breeding traits is still very limited. New advances in the identification of genes and/or QTLs associated with agronomic characters are described in the following articles of this Research Topic.

Relevant agronomic traits in stone fruits include tree architecture. Particularly in peach, vigorous growth and branching used to be controlled by the time-consuming process of pruning or by treating with paclobutrazol, a gibberellins inhibitor. In this context, Cheng et al. have identified seven gibberellin 2-oxidase genes (*PpGA2ox*) in peach that clustered into three subgroups and which ectopic expression in tobacco induces dwarfism and smaller leaves. Moreover, the analysis of the transgenic plants showed that *PpGA2ox* genes deactivate C-19 and C-20 gibberellins (GAs) and that GA3 treatment of shoot tips induces their expression with different time courses depending on the subgroup. These results identify *PpGA2ox* genes as interesting potential targets for fine-controlling endogenous GA levels and, consequently, tree growth in peach. Also related to tree growth, the non-infectious bud failure (NBF) is a serious genetic disorder of unknown cause that affects almond. NBF limits growth and floral bud development causing significant yield losses and it was previously associated with differential DNA methylation patterns. To test this hypothesis, D'Amico-Willman et al. developed and studied an F_1_ interspecific population from a cross between the peach cultivar “40A17” (no-NBF) and the almond cultivar “Carmel” (NBF). Half of the progeny showed NBF symptoms including canopy die-back, erratic branching and rough bark. Genome wide DNA methylation profiling revealed higher methylation levels in NBF hybrids in CG and CHG contexts compared to no-NBF hybrids when aligned to the almond genome but no differences when aligned to the peach genome. Over one hundred thousand differentially methylated regions (DMRs) associated with NBF-exhibition were identified. Nearby genes associated with the 39 most significant DMRs included uncharacterized proteins and transposable elements. Some of these genes showed differential expression patterns linked to differential DNA methylation and represent key targets for almond breeding to mitigate NBF effects.

Along the same line, autotoxicity is a special kind of allelopathy that interferes plant growth and is considered a major factor underlying replanting problem. This phenomenon affects stone fruits in general but particularly peach, where Benzoic Acid (BA) is an autotoxin widely associated with plant growth inhibition. However, the molecular mechanisms controlling peach response to BA stress are mostly unknown. As part of this topic, Shen et al. identified 6,319 differentially expressed genes after BA stress in peach including a number of genes involved in photosynthesis, redox metabolism, stress-response, and transcription control. In addition, up to 74 differentially accumulated metabolites were found in BA-stressed roots including amino acids, fatty and organic acids, and sugars. Co-joint KEGG enrichment analysis indicated that most co-mapped pathways were associated with amino acid and carbohydrate metabolism suggesting that BA stress may disturb carbon and nitrogen metabolism in peach roots. This study provides solid basis for elucidating the autotoxicity response mechanism in peach and for developing strategies to alleviate the replant problem.

Blossom is a central trait in *Prunus* breeding programs due to its major relevance for commercial purposes. To identify genes underlying flowering time control, studies have commonly explored trait-associated QTLs but Shirasawa et al. chosen a distinct approach paying attention to differential gene expression in cherry (*Cerasus* × *yedoensis*). Authors compared RNA-seq data from floral buds and flowers in anthesis stage and results revealed up to seven groups of genes expressed at different times within an interval ranging from 5 weeks before blooming to 2 weeks post-blooming. Based on these data, Gene Ontology terms enrichment analysis supported that molecular mechanisms underlying flowering in cherry involve genes encoding transcription factors, phytohormones, transporters and polysaccharide metabolic enzymes. Interestingly, a statistical model for predicting blooming date was implemented based on gene expression levels at different time points before flowering.

In the field of fruit quality, acidity is a key factor for consumer acceptance, but despite this, its genetic control mechanism is poorly understood. Dondini et al. analyzed QTLs associated with this trait by using an F_1_ apricot population “Lito” × “BO81604311”. For this purpose, titratable acidity, juice pH and fruit contents in malate, citrate and quinate acids were measured in the progeny. QTL mapping was performed on an available SSR-based map enriched with new markers in specific genomic regions. Several major QTLs linked to fruit acidity-related traits (distributed in linkage groups LG4, LG5, LG6, LG7, and LG8) were identified both in “Lito” and “BO81604311”. Some QTLs showed good stability across years and linked markers were used to identify acidity-related candidate genes.

This Research Topic was designed to compile significant advances on *Prunus* targeted breeding based on approaches aimed at identifying new useful genes. Five original articles have been published in this Research Topic that cover some main *Prunus* crops (i.e., peach, cherry, almond, and apricot) and diverse important agronomic traits related to tree growth such as GA homeostasis, autotoxicity stress and non-infectious bud failure as well as blossom and fruit acidity. As a whole, this compilation contains recent contributions in the field based on different approaches that pave the way for implementing DNA-informed breeding in stone fruits.

## Author Contributions

All authors listed have made a substantial, direct, and intellectual contribution to the work and approved it for publication.

## Conflict of Interest

The authors declare that the research was conducted in the absence of any commercial or financial relationships that could be construed as a potential conflict of interest.

## Publisher's Note

All claims expressed in this article are solely those of the authors and do not necessarily represent those of their affiliated organizations, or those of the publisher, the editors and the reviewers. Any product that may be evaluated in this article, or claim that may be made by its manufacturer, is not guaranteed or endorsed by the publisher.
